# Special Issue: “Infection in Honey Bees: Host–Pathogen Interaction and Spillover”

**DOI:** 10.3390/pathogens11010077

**Published:** 2022-01-08

**Authors:** Giovanni Cilia

**Affiliations:** CREA Research Centre for Agriculture and Environment, Via di Saliceto 80, 40128 Bologna, Italy; giovanni.cilia@crea.gov.it

Honey bee health is a very important topic that has recently raised the interest of researchers. In addition, the health of honey bees is strictly related to ecosystem health; therefore, honey bee species act as reservoirs for several pathogens widely spread and able to infect wild pollinators, contributing to their decline. The basis of this Special Issue is to contribute to the knowledge on host–pathogen interactions, in honey bees as well as wild bees.

To explore all possible features of the dynamics of honey bee pathogens, this Special Issue entitled “Infection in Honey Bees: Host–Pathogen Interaction and Spillover” aimed to explore a series of research articles focused on different aspects of honey bee pathogens and their interaction with the hosts. The published papers highlighted this theme at different levels—namely, considering any aspect of the host–pathogen interaction, focusing on different aspects of pathologies, or giving particular attention also to the spillover. All 13 Published articles explored this theme and emphasized the importance of this issue.

Chang et al. compared the genomic sequencing of Sacbrood virus (SBV) strains from the Asian honey bee, *Apis cerana*, and European honey bee, *Apis mellifera*, in Taiwan. Each viral genome encoded a polyprotein, which consisted of 2841 aa in *A. cerana* and 2859 aa in *A. mellifera*, and these sequences shared 95% identity. Compared with the other 54 SBV sequences, the structural protein and protease regions showed high variation, while the helicase region was the most highly conserved. Moreover, 17 amino acids resulted deleted in the viral protein 1 (VP1) region of *A. cerana*, compared with *A. mellifera*. The amino acid difference in the VP1 region might serve as a molecular marker for describing SBV cross-infection [1].

The effects of the application of the commercialized herbal supplements NOZEMAT HERB^®^ and NOZEMAT HERB PLUS^®^ for treating *Nosema ceranae* infection were investigated in 45 selected honey bee colonies. The obtained results reveal that both herbal supplements showed statistically significant activity against *N. ceranae* in infected apiaries. The results suggest a new approach as an alternative therapy to control nosemosis, even if the mechanism of their action is still not elucidated [2].

Nanetti et al. assessed the presence of *Lotmaria passim, Crithidia mellificae*, and replicative forms of deformed wing virus (DWV) and Kashmir bee virus (KBV) in *Aethina tumida*, using specimens collected from *A. mellifera* colonies in Gainesville (Florida, USA), in summer 2017. The replicative forms of KBV have not previously been reported. The results provide evidence of pathogen spillover between managed honey bees and small hive beetles, and these dynamics require further investigation [3].

Ptaszyńska et al. analyzed data collected from honey bees at various time points from anthropogenic landscapes in relation to amplicon sequencing of the *16S rRNA* from bacteria and ITS2 regions of fungi and plants. The differences found between samples were mainly influenced by the bacteria, plant pollen, and fungi. Additionally, honey bees fed with a sugar-based diet were more susceptible to *N. ceranae* and neogregarines, even in cases of co-infection. Healthy honey bees had a higher load of plant pollen and several bacterial groups. Finally, the period when honey bees switch to winter generation is the most sensitive to diet perturbations, and hence pathogen attack, for the entire beekeeping season. Evolutionary adaptation of bees may fail to benefit them in modern anthropomorphized environments [4].

Dechatre et al. proposed models for the prediction of *Varroa destructor* infestation in *Apis mellifera*. The models are based on easy and rapid use of measurable data—namely, phoretic *Varroa* load and capped brood cell numbers. Using these models, beekeepers will be able to either evaluate the risks and benefits of treating *Varroa* or anticipate the reduction in colony performance due to mites during the beekeeping season [5].

Naree et al. evaluated the effects of propolis extract of stingless bee *Tetrigona apicalis* and chitooligosaccharides (COS) on *N. ceranae* infection in giant honey bees *Apis dorsata*. In the infected bees, propolis extracts and COS caused a significant increase in trehalose levels in hemolymph, protein contents, survival rates, and acini diameters of the hypopharyngeal glands. All these changes suggest that both natural compounds could improve the health of infected honey bees [6]

Honey bee virus infections were studied in wild bumblebees, in Croatia. Acute bee paralysis virus (ABPV), black queen cell virus (BQCV), chronic bee paralysis virus (CBPV), and DWV were found in the investigated specimens. BQCV reported a higher prevalence, followed by DWV, ABPV, and CBPV, respectively. Moreover, BQCV and DWV strains showed a high similarity of 95.7 % and 98.09% nucleotide identity, respectively, with previously identified honey bees in Croatia and Slovenia, providing insights into highly diverse strains circulating in wild bees [7].

Power et al. investigated the histopathological features of 25 symptomatic and asymptomatic honey bees naturally infected with DWV. The results showed degenerative alterations of hypopharyngeal glands (in 19 specimens) and flight muscles (in 6 specimens) in symptomatic samples, while in 4 asymptomatic samples, evidence revealed an inflammatory response in the midgut and hemocele. All these findings suggested a possible pathogenic action of DWV in both symptomatic and asymptomatic honey bees, improving their immune response by keeping the virus under control in asymptomatic honey bees [8]. 

In 2017 and 2018, clinically healthy workers of bumblebees and honey bees were collected on flowers in four different areas of Slovenia to assess the spillover of honey bee pathogens. The results evinced a prevalence of 58.5% for BQCV, 24.5% for SBV, 17.0% for *Crithidia bombi,* 16.3% for *Nosema bombi*, 15.6% for Lake Sinai virus (LSV), 15.0% on *Apicystis bombi*, 8.8% for ABPV, 8.2% on *N. ceranae*, and 6.8% for DWV. The study confirmed that several pathogens are regularly detected in both bumblebees, suggesting important spillover events [9].

Cappa et al. highlighted the association between bee decline and the type of land surrounding the apiary. The authors developed a risk map to identify the areas with the highest risk of bee decline in Lombardy. The apiaries were considered “declined” if they reported at least one event of decline or tested positive for plant protection products, while the apiaries were “not declined” if they did not report any events of bee decline during the study period. Out of 14,188 apiaries analyzed, 80 were considered declined. Furthermore, the risk maps highlighted that the probability of apiary deterioration increases by 10% in orchards and 2% in arable lands. This information can be used by Italian Veterinary Services as a predictive measure for planning prevention and control activities [10].

Braglia et al. investigated the control of *N. ceranae* by using several compounds. The results showed that some of the ingredients administered, such as acetic acid at high concentration, p-coumaric acid, and *Saccharomyces* sp. strain KIA1, were effective in the control of nosemosis. On the other hand, wine acetic acid strongly increased the *N. ceranae* amount. The effects of all tested compounds can be investigated in more detail, especially to improve honey bee health [11]. 

Alonso-Prados et al. investigated the possible underlying causes behind the poor health of a professional *A. mellifera iberiensis* apiary located in Gajanejos (Guadalajara, Spain). The case report highlighted several factors that potentially favor colony collapse, including pathogen infections and accumulation in the beebread of coumaphos and tau-fluvalinate (acaricides commonly used to control *Varroa destructor*). The high level of acaricides and unusual climatic conditions of the year suggested a possible increase in vulnerability to infection by *N. ceranae* and the consequentially collapse events. This case report highlighted the importance of evaluating all possible factors in future monitoring programs, to adopt adequate preventive measures aimed to guarantee the health and fitness of bees [12].

Finally, in a systematic review, the pathogen spillover from honey bees to other arthropods was analyzed by Nanetti et al. The systematic review amassed and summarized spillover cases having in common *Apis mellifera* as the maintenance host and some of its pathogens. The collected data were grouped by final host species and condition, year, and geographic area of detection and the co-occurrence in the same host. In total, 81 articles in the time frame of 1960–2021 were analyzed. The reported spillover cases were evaluated in a wide range of hymenopteran species, generally sharing the same environment with the honey bees. Moreover, the honey bee pathogens are able to infect non-hymenopteran arthropods, such as spiders and roaches, which are either likely or unlikely to live near honey bees. The plasticity of bee pathogens and ecological consequences of spillover necessitate an approach that emphasizes bee health as well as the health of the ecosystem, fully implementing a One-Health outlook [13].
